# A Perspective on Research on Dishonesty: Limited External Validity Due to the Lack of Possibility of Self-Selection in Experimental Designs

**DOI:** 10.3389/fpsyg.2017.01566

**Published:** 2017-09-12

**Authors:** Petr Houdek

**Affiliations:** ^1^Faculty of Social and Economic Studies, Jan Evangelista Purkyně University in Ústí nad Labem Ústí nad Labem, Czechia; ^2^Faculty of Business Administration, University of Economics, Prague Prague, Czechia; ^3^Center for Behavioral Experiments Prague, Czechia

**Keywords:** dishonesty, external validity, self-selection, sorting, organization, laboratory experiment, field experiment, ethics

## Abstract

The aim of this perspective article is to show that current experimental evidence on factors influencing dishonesty has limited external validity. Most of experimental studies is built on random assignments, in which control/experimental groups of subjects face varied sizes of the expected reward for behaving dishonestly, opportunities for cheating, means of rationalizing dishonest behavior etc., and mean groups’ reactions are observed. The studies have internal validity in assessing the causal influence of these and other factors, but they lack external validity in organizational, market and other environments. If people can opt into or out of diverse real-world environments, an experiment aimed at studying factors influencing real-life degree of dishonesty should permit for such an option. The behavior of such self-selected groups of marginal subjects would probably contain a larger level of (non)deception than the behavior of average people. The article warns that there are not many studies that would enable self-selection or sorting of participants into varying environments, and that limits current knowledge of the extent and dynamics of dishonest and fraudulent behavior. The article focuses on suggestions how to improve dishonesty research, especially how to avoid the experimenter demand bias.

## Introduction

Behavioral ethics is a rapidly growing field investigating numerous factors related to dishonesty. Since people typically hide their dishonest behavior, it is difficult to measure the factors’ causal influence only by data observed in the real world (Zitzewitz, 2012; Pierce and Balasubramanian, 2015; Houdek, 2017a). Studies therefore use controlled laboratory and field experiments to be able to identify the participants’ behavior based on the experimental manipulations they face: sizes of the expected reward for behaving dishonestly, opportunities for cheating, means of rationalizing dishonest behavior, in-group v. out-group mentality etc. (Gino et al., 2009; Rosenbaum et al., 2014; Irlenbusch and Villeval, 2015; Shalvi et al., 2015; Jacobsen et al., 2017; Novakova et al., 2017).

The experimental evidence is built on random assignments into control and experimental groups, and mean groups’ reactions are observed. However, people strive (not) to find themselves in a situation with a greater/lower motivation/opportunity to cheat. If people can opt into or out of diverse real-world environments, an experiment aimed at testing factors influencing real-life degree of dishonesty should permit for such option (Lazear et al., 2012). The behavior of such (self-)selected groups of marginal subjects would probably contain a larger level of (non)deception than the behavior of average people (for effects of sorting in various experimental designs, see Orbell and Dawes, 1993; Bohnet and Kübler, 2005; Cadsby et al., 2007; Dohmen and Falk, 2011; for a similar problem of selective attrition in web-based studies see Zhou and Fishbach, 2016).

Laboratory experiments are frequently criticized for their weak external validity. Tasks in which the influence of a given factor on behavior is tested are necessarily artificial and the findings may not generalize to real-world settings. In laboratory experiments, there is scrutiny unparalleled in the field, lack of credible anonymity, not completely controlled context, low stakes, self-selection into the participation in an experiment, and restricted choice sets and time horizons (Levitt and List, 2007). Nevertheless, studies comparing the behavior of subjects in a laboratory and then in field conditions show some correlations (Potters and Stoop, 2016; Dai et al., 2017; Hanna and Wang, 2017). In this perspective article, I will elaborate another aspect limiting the external validity of current experimental studies: abstracting from institutional (organizational) dynamics.

## The Self-Selection Problem

An experimental approach measuring a causal effect of an observed factor just by randomized assignments of participants into experimental/control groups has a limited external relevance due to the assumption that the influence of sorting is negligible in real life. But people tend not to be randomly assigned to a contest, profession, team, a certain boss, or randomly get in charge of a process (although it can happen). Rather, they strive to work in environments they perceive as suitable for themselves or are assigned into environments they fit into.

Workers who are more gregarious tend to be employed in jobs that involve more social interactions (Krueger and Schkade, 2008), women shy away from competition and men embrace it (Niederle and Vesterlund, 2007), entrepreneurs have greater self-esteem and tend to have been engaged (as teenagers) in more disruptive, illicit activities (Levine and Rubinstein, 2017), single parents with children are less willing to work in occupations with a relatively high accident rate than people without children (DeLeire and Levy, 2004), etc. (Azmat and Möller, 2009; Leuven et al., 2011; John and Thomsen, 2014). As Lazear, Malmendier, and Weber put it: “an experiment with randomly selected individuals might reveal that a significant portion of subjects suffer from acrophobia. But sorting and voluntary selection ensure that those who build skyscrapers are unlikely to be among the sufferers” (p. 1, working paper version of Lazear et al., 2012). Moreover, the selection of a fitting environment runs on everyday basis. People consciously strive not to succumb to the actions they want to avoid; as “cutting up one’s credit cards, only taking a fixed amount of cash when heading out to party for a night, buying junk food in small packages rather than buying in bulk, not keeping alcohol in the house, brushing one’s teeth earlier in the evening to avoid late night snacking” (Bryan et al., 2010, p. 675).

It can therefore be expected that in a market or organizational environment, there will be a strong sorting of employees into different environments where dishonest behavior is more or less possible, tolerated or expected. For instance, in a laboratory study (Dana et al., 2006) subjects could participate in the Dictator Game and divide 10 dollars, or choose 9 dollars as an “exit option,” where the co-player did not learn about the option of the game. Since they could have kept 10 dollars in the game, selection of the exit option by a third of the participants has shown that it is psychologically costly for them to choose to be in a situation where they have an opportunity to behave unfairly. Similarly, Fehrler and Kosfeld (2014) showed in a laboratory labor market experiment that some participants self-select into treatments where they could give up monetary rewards for the option to generate a donation to their preferred NGO; see also (Brekke et al., 2011).

An experiment which uses randomized assignment of participants can come to a potential conclusion that an observed factor does not influence dishonesty. Such conclusion is of course causally valid; however, it does not imply that this factor does not have an observable influence in a comparable real-world situation where people can self-select into their preferred environments. A now famous study (Mazar et al., 2008) tested the influence of the probability of detection on the rate of cheating in a mathematical task. Participants in the control group handed in their sheets for checking, while participants in the experimental groups were instructed to partially or fully destroy the documents proving their cheating (so that the risk of detection was varied). Some collected their reward by themselves so that the experimenter not only did not check their performance, s/he also did not encounter them during the reward collection. The study has identified that the participants cheated to a low extent, and did not find the rate of cheating to increase with the extent of document destruction.

Comparable results reporting relatively low rates of cheating were found by most studies investigating several other factors that could modulate cheating, such as self-justification availability, moral licensing, perception of unfair treatment, beneficiary identity, etc. (Shalvi et al., 2011; Houser et al., 2012; Lewis et al., 2012; Fischbacher and Föllmi-Heusi, 2013; Jiang, 2013; Abeler et al., 2014). However, let us imagine that people would apply for a job whose description specifically stated that their reward would depend only on what amount of money they demand, while no one will check upon them and there would be no proof of their actual work. How extensive would cheating be in this case?

It is not enough to use heterogeneous groups of participants in experiments (Henrich et al., 2010), it is necessary to allow participants to choose their preferred environment, as such dynamics apply in the real world. If a study strives to have relevance for the organizational or market sphere, it should assume that there is a difference between results achieved by randomly assigning and by enabling a self-selection of participants into the groups; it should therefore compare the size of the effect in the design of random assignments and the self-selection design (Bless and Burger, 2016). The behavior of specially selected people will be different from the behavior of a group of randomly selected individuals.

One of the mere handful of studies, which directly tested the effect of self-selection into an environment enabling cheating, is by Gino et al. (2013). Their participants could cheat in a matrix task (Mazar et al., 2008) and received a reward if their performance was better than the performance of another (randomly chosen) participant from the same experiment session. The study explored several conditions: mandatory-regulation condition, where the performance of all participants was verified; a no-regulation condition, where all participants were free to report any score, and none of the reported scores are verified; and two conditions with voluntary-regulation conditions, in which participants chose whether to have their scores verified. The study concludes that opting for no regulation induces greater dishonesty. Similar results were reported by another study (Faravelli et al., 2015); although it did not enable selection of environments where cheating is more or less possible, participants could select the method of rewarding. Productivity and cheating were tested, again in several matrix tasks, and the participants could then choose to be rewarded either in a piece rate scheme or a winner-takes-all tournament (the tournament winner receives a higher payoff than in the piece rate scheme, while the tournament loser receives nothing). The study found that dishonest subjects were more likely to select themselves into the winner-takes-all tournaments.

## The Problem of Institutions in Self-Selection

Another aspect of the low external validity is the lack of long-term reputation concerns (or career outlook in the organizational context) in the random assignment design. The choice of where and how to work is, up to some extent, public information. The fact that an employee would choose or pursue a process or environment where they can behave dishonestly [or where such stereotype exists (Vranka and Houdek, 2015)] can threaten their reputation, so they would not choose it. This aspect of sorting could limit the prevalence of environments where it is possible to cheat.

Nevertheless, the opposite dynamics can be true in other cases; in some positions, environments or cultures, it can be expected or tolerated for an employee to be morally flexible (Banerjee et al., 2015; Grieser et al., 2016; Zeume, 2016; Karpoff et al., 2017). As the following studies show, if people know that they are in such an environment, they generally prefer norms allowing dishonesty or collaborators willing to engage in dishonest behavior, e.g., in corrupt countries, managers with a positive regard of corruption are preferred (Mironov, 2015). Professionals in high-salesmanship occupations (e.g., sales, advertising, investment banking) engaging in higher levels of deception are seen as more competent and worthy of competence-based trust (Gunia and Levine, 2016).

Now let us imagine that employees of a given firm learn that it is very likely that they will face situations where they would be compelled to act dishonestly more frequently. Honest employees would likely start leaving the firm, while dishonest or morally flexible ones would stay; e.g., a study (Schweitzer et al., 2004) implies that individuals with ambitious goals incline to widespread cheating. It can therefore be expected that people who tend to behave dishonestly would stay on certain positions or be promoted to them. Then it would be in the interest of a manager of the firm to select employees or colleagues more willing to act dishonestly (because they would be rewarded based on their willingness toward this behavior). It can again be expected that he or she would set the rules so that people also inclined to behave dishonestly would apply. For instance, the employees might be compelled to lie. By lying, they would not only reaffirm their loyalty to the manager or firm, but would need to adapt their subsequent behavior to their lies for their (cognitive) consistency.

In the organizational sphere, these effects would accumulate, and selection of dishonest people and rules supporting dishonesty would arise [certainly, even in an environment where dishonesty strives, certain norms will be required and respected (Leeson, 2009; Skarbek, 2014)]. The expected outcome is based on the theory of moral consistency (Cornelissen et al., 2013), or the organizational theory stating that norms people adhere to and behave according to get homogenized (Garrett et al., 2014). In an environment where honesty is weak, dishonest people prevail and/or people prefer norms enabling them to cheat and they therefore behave even more dishonestly (Weisel and Shalvi, 2015; Gächter and Schulz, 2016). As Faravelli et al. (2015, p. 161) found out: “Once an individual decides to lie, or to increase her level of dishonesty, the relative benefit of honestly produced output suddenly falls. Moreover, such benefit decreases even further if one expects others to lie…”. These predictions stand in contrast to theories of moral licensing, which assume that people strive for balance in their moral self-image, and behave more honestly after previous dishonest behavior, and vice versa (Blanken et al., 2015).

As far as I know, there has been no experimental study testing how is the choice of an environment or process influenced by future expectations of (dis)honest behavior. However, I do not deny that other factors play a role in the outlined dynamic and that they could influence the prevalence of dishonest behavior more than the self-selection or selection of rules enabling dishonesty alone (Treviño et al., 2006; Burks and Krupka, 2012; Zhang et al., 2014). Furthermore, the random assignment method sometimes cannot be substituted, i.e., in studies investigating neuronal mechanisms beyond dishonest decision-making (Garrett et al., 2016; Maréchal et al., 2017).

## Experimenter Demand and Conclusion

In this article, I argue that the external validity of studies on dishonesty is limited, since they neglect the influence of self-selection and sorting of people into preferred environments. If the real world enables people to choose a certain type of environment, while experimental studies only use random assignments into control and experimental groups facing different situations, then the experimental evidence has only a limited relevance. Some environments attract crooks, different environments appeal to honest people. Some managers may prefer morally flexible personnel, who will quickly become accustomed to dishonesty, others try to avoid such people. We can expect that factors such as self-justification availability, moral licensing or in-group vs. out-group mentality would have largely greater or lesser impact on dishonest behavior than experimental studies suggest today, if sorting was enabled. Moreover, depending on various self-selected groups, the effect of a particular factor may increase or decrease over time. However, studies with repeated or enduring manipulations are also rare (e.g., Fisman and Miguel, 2007; Barr and Serra, 2010).

Reflecting the sorting of certain people into environments enabling cheating could point toward a greater importance of personality traits (moral character) that could influence the eventual extent of (dis)honest behavior more than situational factors (Cohen et al., 2014; Griffin et al., 2016). For instance, REVISE measures for supporting honest behavior (Ayal et al., 2015) include situational measures such as: creating cues that increase the salience of morality and decrease ability to justify dishonesty; restricting anonymity; and helping to bridge the gap between abstract moral values and actual behavior. However, if we consider that people planning on behaving dishonestly could avoid these measures in the real world (e.g., they will not work for a firm implementing them), they could show an enormous impact in some environments and they will play at most a very limited role in others (Houdek, 2017b).

Nevertheless, a greater usage of experimental designs enabling self-selection is not a panacea in achieving external validity. Despite utilizing self-selection designs, the results’ validity will remain narrow. One of the little-discussed limitations of laboratory experiments, lowering their external validity, is the effect of experimenter demand. “Experimenter demand effects… refer to changes in behavior by experimental subjects due to cues about what constitutes appropriate behavior (behavior ‘demanded’ from them)” (Zizzo, 2010, p. 75). Participants in a laboratory experiment are not necessarily sure what is the suitable descriptive norm and behave due to their preferences, but sometimes the experimenter’s demand is clearer, and they can try to accommodate it; for instance, if participants know that they are to destroy all documents revealing possible cheating in front of the experimenter, the experimenter likely expects them to cheat (Mazar et al., 2008). On the other hand, if experimenters in a study (Bucciol and Piovesan, 2011) just told subjects not to cheat, the rate of cheating dropped by 16%. If we enable participants to self-select into environments with or without the possibility of cheating, the aim of the laboratory study can also be transparent and they could behave in a “demanded” way.

However, many studies do not conduct (or do not refer about it) a thorough post-experimental survey to find whether the participants guessed the meaning of the experiment or to what extent they accommodated the expected demands of the experimenter. Therefore, the impact of experimenter demand on observed behavior is unclear [nevertheless, in other experiments, participants are unable to discern “obvious” treatments, or do not admit so (Desai and Kouchaki, 2017)].

One approach how to limit the impact of experimenter demand in identifying the effect of the tested factor is to conduct an experiment where participants do not know they are a part of an experiment or think that they are participating in an experiment investigating another aspect of human behavior (Levine et al., 2010; Zizzo, 2010).

A clear example of a study enabling self-selection of participants in a field experiment is by DellaVigna et al. (2012). They tested the willingness of households to contribute to charities and their design included the option of self-selection, since some households were informed about the time the fund-raiser would come and they could avoid the occasion; other households could check a “Do Not Disturb” box on the flyer if they did not want to be disturbed (in baseline treatment, fund-raisers contact households in the usual door-to-door manner without a flyer). The study found the share of households opening the door was 9% lower after receiving information about the time of a fund-raiser visit and 23% lower after receiving the flyer with a “Do Not Disturb” box. Moreover, the flyer with “Do Not Disturb” box reduces giving by 28 to 42% (depending on various target charities). It was shown that a situation where one should give to others, places people under social pressure to give; but some people want to avoid such situations.

Similar studies are necessary also in the realm of dishonesty research, so that it is possible to estimate how (dis)honestly people really behave if they can choose their preferred environment.

## Author Contributions

The author confirms being the sole contributor of this work and approved it for publication.

## Conflict of Interest Statement

The author declares that the research was conducted in the absence of any commercial or financial relationships that could be construed as a potential conflict of interest.
